# Can Anaerobic Soil Disinfestation (ASD) be a Game Changer in Tropical Agriculture?

**DOI:** 10.3390/pathogens10020133

**Published:** 2021-01-28

**Authors:** A. K. Hasith Priyashantha, Renuka N. Attanayake

**Affiliations:** 1 Department of Plant and Molecular Biology, University of Kelaniya, Kelaniya 11600, Sri Lanka; Priyashanthahasith@gmail.com; 2 Department of Multidisciplinary Studies, Faculty of Technology, Eastern University, Batticaloa 30376, Sri Lanka

**Keywords:** soil-borne pathogens, chemical fumigation, anaerobic soil disinfestation, ASD, C source

## Abstract

Anaerobic soil disinfection (ASD) has been identified as an alternative soil-borne pathogen control strategy to chemical fumigation. ASD involves the application of an easily liable carbon source followed by irrigation to field capacity and maintenance of an anaerobic condition for a certain period. A literature search undertaken on ASD found that more than 50 comprehensive research projects have been conducted since its first discovery in 2000. Most of these studies were conducted in the USA and in the Netherlands. Though the exact mechanism of ASD in pathogen control is unknown, promising results have been reported against a wide range of pathogens such as fungi, nematodes, protists, and oomycetes. However, it is interesting to note that, except for a few studies, ASD research in the developing world and in the tropical countries has lagged behind. Nevertheless, with soil quality depletion, reduction in arable lands, and exponential population growth, a drastic change to the current agricultural practices should be adapted since yield gain has reached a plateau for major staple crops. Under such circumstances, we identified the gaps and the potentials of ASD in tropical agricultural systems and proposed promising biodegradable materials.

## 1. Introduction

Crops are often attacked by various plant pathogens, plant-parasitic nematodes, insect pests, and weeds causing great economic losses around the world. Among diverse groups of plant pathogens, soil-borne phytopathogens pose a great threat to crop production [1,2,3]. Although soil is a home for billions of living organisms (both macro and microorganisms), they must face a multitude of challenges such as flood, drought, and agricultural practices. However, soil-borne pathogens can survive under these challenges and cause serious crop damage around the world. For example, waterlogged agricultural fields may be unfavourable for many organisms but favourable for root-infecting fungi and oomycetes such as *Pythium* and *Phytophthora* spp. [4,5,6]. Although drought conditions are unfavourable for most of the organisms, soil-borne pathogen species such as *Fusarium* spp. and *Verticillium* spp. [5] manage to cause severe infections. Hence, soil-borne phytopathogens show a great deal of evolutionary adaptations. They can survive in the soils for many years in the absence of host plants through the formation of resistant structures such as microsclerotia (*Verticillium* spp.), sclerotia (*Sclerotinia* spp.), chlamydospores (*Fusarium* spp.), or oospores (*Phytophthora* spp.) [7,8,9,10]. Microsclerotia and sclerotia have the same anatomical structure, consisting of outer melanized parenchyma cells and inner colorless medullary cells, and are asexual in nature. Chlamydospores are thick-walled asexual survival structures whereas oospores are thick-walled sexual structures with food reserves for better survival. These structures may be melanised or non-melanised. Melanisation of survival structures has several evolutionary advantages such as protection from UV radiation, successful penetration during infection, long-term survival, growth, and development [11,12]. Wilhelm [13] found the persistence of microsclerotia of *Verticillium alboatrum* for 14 years in soil, which were viable even after the exposure to desiccation at high temperatures. Ben-Yephet et al. [14] reported the survival of sclerotia of soil-borne *Sclerotinia sclerotiorum* declined after an outbreak of lettuce drop, nevertheless, about 5.5% were viable even after seven years. Babadoost and Pavon [15] assessed the survival of *Phytophthora capsici* oospores in the soil in Illinois (USA) and found three to four years of survivability. Apart from soil-borne fungal plant pathogens, plant-parasitic nematodes have been recognized as another group of challenging pathogens to manage [16].

Besides, each plant can be infected by several pathogen species and the complex nature of the soil environment, it is difficult to control diseases caused by soil-borne pathogens. Hence, successful control of soil-borne pathogens is a major challenge due to inherent difficulties of disease prediction, early detection, and accurate diagnosis [2]. Some modern crop production systems are based on raised-bed, plasticulture, and limited or short crop rotation-lengths, probably with the unavoidable application of broad-spectrum soil fumigants to manage pests and diseases [1]. Since the mid-20th century, synthetic chemicals have been used to control many plant diseases including a broad spectrum soil fumigant, methyl bromide (MeBr) [17,18,19]. Since then, MeBr has been heavily applied worldwide primarily to control soil-borne pathogens as well as the nematodes [20]. For example, five million kg of MeBr were used only in California in the year 2000 [21]. MeBr has been identified as a stratospheric ozone-depleting component by the U.S. Environmental Protection Agency (EPA) and the United Nations Environment Program (UNEP). Bolstered by the 1994 UNEP Montreal Protocol on Substances that Deplete the Ozone Layer, MeBr was identified as a major ozone-depleting compound [22]. Thereafter, MeBr was completely banned by the 1 January 2005 with few exceptions [19,21,23,24].

Alternative synthetic fumigants such as 1,3-dichloropropene, 1,3-D, chloropicrin, trichloronitromethane, methyl isothiocyanate, allyl isothiocyanate (AITC), and dazomet were tested and applied by the farming communities around the world yet were poorly accepted due to geographic limitations, reduced efficacy, and regulatory constraints [25,26,27]. Moreover, many criticisms have been generated from the public and from the scientific communities against the use of such chemical soil disinfestation methods due to their toxicity on humans and undesirable effects on non-target organisms such as beneficial microflora, groundwater pollution, and development of resistance [19,28,29,30,31,32].

Therefore, farmers were compelled to use non-chemical approaches. Traditionally a number of environmental friendly approaches such as mixed cropping, crop rotation, resistant cultivars/selective breeding, application of biocontrol agents, flooding, solarisation, steaming, pasteurisation, hot water treatment, and bio-fumigation have been applied by farmers around the world to mitigate soil-borne diseases [19,33,34,35]. Nevertheless, these applications were not as popular as chemical fumigants due to several limitations [19]. Application of mixed cropping systems may be helpful in increasing the crop yield while addressing some of the soil-borne pathogen problems [36], yet it is not always economically feasible when the rotation is done with low economical value crops [35]. Although selective breeding shows some level of effectiveness against soil-borne pathogens, host resistance breakdown has been reported, and no completely resistant cultivars are available for all the crops [35]. Another option would be the use of biocontrol agents, however, these are highly specific for particular pathogen species if not for strains, and effectiveness is greatly dependent on the environmental factors [37]. Similarly, other non-chemical approaches have their own disadvantages, hence there have been limited applications [33,36,38,39,40,41].

### Anaerobic Soil Disinfestation (ASD)

To minimize the above drawbacks of chemical and non-chemical methods of soil-borne pathogen control, researchers found alternative methods, and one such promising approach is anaerobic soil disinfestation (ASD), also called biological soil disinfection (BSD) or reductive soil disinfection (RDS). This method was first described independently by researchers in Japan [42,43] and in the Netherlands [44] and was later adapted to the USA [45] to control soil-borne pathogens in strawberry and vegetable fields. Thereafter, researchers around the world started applying this method, showing a great potential to control various soil-borne phytopathogens [44,45,46,47,48,49,50,51,52,53,54,55]. 

The method is characterized by non-chemical pre-plant control of soil-borne phytopathogens using few simple steps [29,56]. The first step of ASD is the incorporation of organic amendments (usually an easily labile carbon source) to the topsoil. The soil is later wetted to field capacity and covered with a clear (preferably black) and gas-impermeable polyethylene sheet for a defined period of time to maintain an anaerobic condition [57]. The effectiveness of ASD has been evaluated against soil-borne diseases such as potato brown rot [46], spinach and tomato wilt diseases [48], *Prunus* [58] and apple replant disease [50], Fusarium wilt of banana [59], root and crown rot diseases of pepper [60], etc., with promising results. ASD has now become popular in organic agriculture worldwide and is practiced under greenhouse and field conditions as well [47,51,61]. There is some evidence that ASD also can contribute to the development of disease-suppressive soils [57]. The objectives of this review were to thoroughly analyse all the studies conducted on ASD since its first discovery two decades ago, to discuss the current trends to identify the gaps of ASD research, especially emphasizing future research directions, and to discuss the potential use of ASD in the tropical agricultural systems.

## 2. Data Collection and Analysis

**Figure 1:**
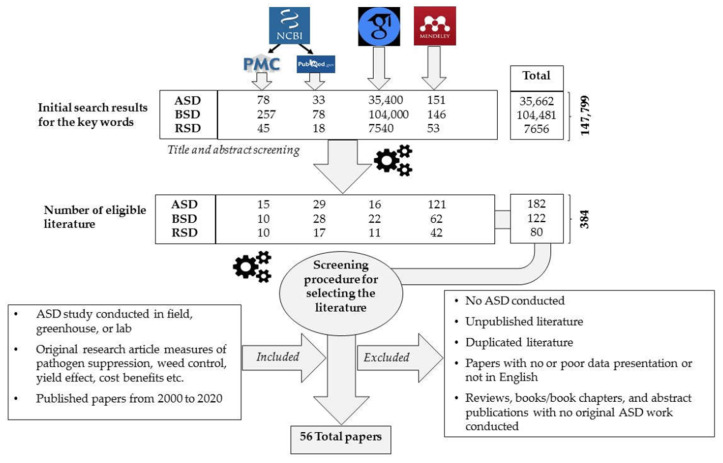
Literature selection procedure. ASD—anaerobic soil disinfection; BSD—biological soil disinfection; RSD—reductive soil disinfection.

A thorough literature search was conducted from National Center for Biotechnology Information (NCBI), Google Scholar databases, and Mendeley referencing tool using the keywords anaerobic soil disinfection, biological soil disinfection, and reductive soil disinfection to filter studies conducted on these aspects during the past two decades. In this initial search, a total of 147,799 results were obtained. However, most of the outcomes were not directly relevant to our objectives, and the selection pipeline is shown in Figure 1. This literature was further analysed to extract information on study region, year, targeted pathogen, weed control, C source used, type of crop, duration of anaerobic period, type of mulch, crop yield improvement, etc. Review papers and duplicated, salami (fragmented publications) and irrelevant publications were excluded from the analysis. Finally, 56 complete, directly relevant, and original research publications originated in nine countries were included in the analysis. Some of these research papers have described ASD effect on more than a single pathogen species and in such instances, they were considered as two or more studies depending on the number of targeted pathogens. Therefore, final analysis was based on 109 studies published in 56 research papers. 

## 3. Trends and Gaps in Application of ASD

### 3.1. Geographical Projection

**Figure 2:**
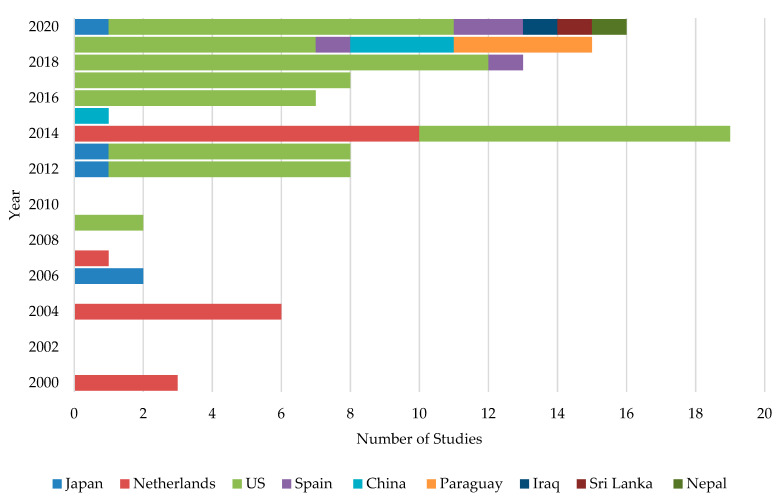
International distribution of ASD studies conducted in each year.

When ASD was first introduced in Japan, it was initially suggested to be used with organic materials such as wheat bran, molasses, rice straw, and rice bran specifically at 1 to 2 tons per 0.1 ha, followed by flooding and plastic film covering of the soil surface [42]. In Netherlands, Blok et al. [44] carried out a two-year ASD field experiment in 1994 and 1995 using fresh broccoli or grass (3.4 to 4.0 kg fresh weight m^−2^) as C sources. They came up with the landmark finding that there was a significant control of soil-born fungal pathogens: *Fusarium oxysporum*, *Rhizoctonia solani,* and *Verticillium dahliae.* The study was published in 2000 and concluded that this novel method could control a wide range of phytopathogens [44]. Based on the published data, it is clear that the initial development of ASD was restricted to the Netherlands and Japan and was later expanded to the USA. However, beyond this point, ASD research showed slow progress until 2014, in which the number of publications were more than doubled (Figure 2). During the past few years, several other countries have also attempted to mitigate soil-borne diseases through ASD.

It is interesting to note that almost all the studies have been restricted to nine countries—primarily the USA (63.3%) followed by the Netherlands (18.3%) and Japan (4.6%). Spain, China, and Paraguay shared about 11.1% of ASD studies equally. The rest of the studies were conducted in Iraq, Sri Lanka, and Nepal, where only one study has been conducted in each country. 

### 3.2. Application of ASD to Control Pathogens, Weeds, and Effect on Crop Yield

**Figure 3:**
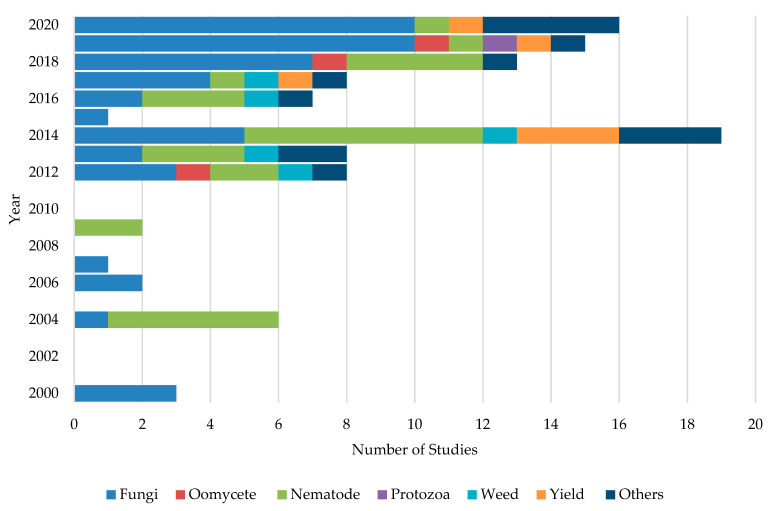
Different ASD studies conducted during the past two decades targeting each group of soil-borne pathogens and other aspects of crop production systems.

Initially, ASD studies were applied to control soil-borne phytopathogenic fungi [44]. Later on, it expanded towards control of nematodes, oomycetes, weeds, and protozoans. However, studies on ASD targeting soil borne-fungi have been extensively carried out mainly due to their broad host range, enormous losses in crop yield and quality, worldwide distribution, management difficulties, and extensive use of synthetic fungicides [62]. For example, 46.8% of the studies were concentrated on the control of fungal pathogens followed by 26.6%, 5.5%, and 4.6% of studies dedicating to testing the effects on nematodes, yield increase, and weed control, respectively. Moreover, about 12.8% of studies have been carried out with different aspects such as evaluating the effect of ASD on soil microflora and cost benefits of the application of ASD. Figure 3 shows the number of studies conducted in each year targeting soil-borne pathogens and other aspects. A majority (63%) of ASD studies were carried out under field conditions. About 35.1% of studies were performed as greenhouse or growth chamber experiments, while about 1.9% of the studies were conducted as lab experiments.

About 28.7% of the ASD studies were targeted to control tomato pathogens while 13%, 9.3%, and 7.4% of the studies were targeted to control strawberry, potato, and bell pepper pathogens, respectively. About 12.9% of the studies did not report the target crop or the intended pathogen to control. The remaining studies were carried out to control soil-borne pathogens associated with lettuce, mustard green, spinach, carrot, cabbage, cauliflower, eggplant, lily bulb, and common bean production fields. Studied organisms included pathogenic fungi: *Fusarium oxysporum, Verticillium dahlia, Colletotrichum coccodes, Sclerotinia sclerotiorum,* and *S. rolfsii,* nematodes: *Meloidogyne hapla, M. incognita* and *Pyrenochaeta lycopersici*, oomycetes: *Phytophthora capsici* and protist: *Plasmodiophora brassicae* etc.

### 3.3. C Source Dependency of ASD

The effectiveness of ASD predominantly depends on the selection of C source, C:N ratio, rate of its application, and anaerobic period. However, soil temperature, water holding capacity of soil, and climatic conditions should also be considered before implementing ASD [63,64,65]. C sources should be easily applicable, readily available/locally available, easily degradable, affordable, and able to control a broad spectrum of phytopathogens [65]. Careful selection of C source seems to play the key role in ASD since several studies have shown the emission of volatile compounds with strong pathogen inhibitory activities. Use of *Brassica juncea* cv. Pacific Gold seed meal (seed meal is a waste product of the oil extraction process) as the C source caused the release of isothiocyanates, alcohols, organic acids, organic sulphides, and esters, while application of orchard grass residues released organic sulfides, ketones, organic acids, and hydrocarbons. Similarly, the application of rice bran-treated soils emitted a spectrum of volatile compounds containing organic acids, alcohols, and esters [50,66]. Mahalingam et al. [55] conducting a gas chromatography-mass spectrometry (GC-MS) analysis of cabbage and leek cull piles reported the presence of antifungal volatiles. 

Moreover, fresh and dried plant materials and composted broiler litter have been tested in multiple studies as the C source in ASD-based studies [54,65,67]. Ethanol has been incorporated as a C source in controlling phytopathogens due to the inefficiency of some of the commonly used C sources. As an example, Momma et al. [68] found that the use of wheat bran alone is not effective in controlling *Fusarium oxysporum* infection of tomato. However, once the soil is saturated with 1% ethanol solution (ethanol medicated ASD treatment), high levels of suppression of *F*. *oxysporum* were observed. Hewavitharana et al. [53] also reported that ethanol (10%) mediated ASD effectively controlled apple root infection caused by *Rhizoctonia solani* AG-5 and *Pratylenchus penetrans.* In addition, it has been reported that ethanol temporarily increased the anaerobic bacterial population [68]. A summary of C source, application rate, target pathogen group, and optimum temperature along with the reference are shown in the Table 1 below.

### 3.4. ASD against Nematodes

Plant pathogenic nematodes are another group of organisms posing a severe threat to worldwide agriculture, especially in developing countries. With the limited availability of nematicides, negative impacts of available chemistries and resistance development have always demanded alternative management options [70]. ASD has shown promising results in controlling plant-parasitic nematodes in several studies conducted in the USA [71,72] and in the Netherlands [52]. A study conducted by Mazzola et al. [73] in the USA found successful control of *Pratylenchus penetrans* in strawberry fields when *Brassica juncea* seed meal was used as the C source, whereas Testen and Miller [74] reported reduction of *M. hapla* when wheat bran and molasses were used. Similarly, tomato plant residues with fresh sheep manure were effective in controlling *M. incognita* [75]. Korthals et al. [52] reported that ASD was more effective and longer-lasting against *P. penetrans* and *V. dahliae* than chemical control, and Di Gioia et al. [76] also reported ASD was effective as chemical soil fumigation against *Meloidogyne* sp. However, it should be noted that the selection of C source should be done carefully, and targeted organism should be taken into account. As an example, Korthals et al. [52] demonstrated that *B. junceae* leaf incorporation (no anaerobic condition was imposed) increased *P. penetrans* density in soil. 

### 3.5. Effect of ASD on Weed Control and Yield

Weed control is one of the major requirements, especially in the tropics, where year-round cultivation is practiced, favouring accumulation of weed seed banks. Each year, billions of dollars are spent on herbicide development and applications. Application of one of the most controversial yet most effective herbicides, glyphosate, has dramatically increased in the last two decades and by the end of 2014, 8.6 billion kg of glyphosate had been applied globally [77]. However, recently, there are serious concerns over the use of glyphosate and its negative impacts on human and environmental health. Mixed results have been presented in terms of the effect of ASD on weed control. While Shennan et al. [66] and Guo et al. [64] reported a low success rate or failure of weed control during ASD with either rice bran or molasses as C sources, McCarty et al. [49] reported effective weed control with cereal rye and mustard/arugula in Tennessee, USA. Lamers et al. [78] emphasized that green manure crops should be amended at least at 40 t ha^−1^ rate to achieve weed control in the Netherlands. Although not as effective as chemical treatments, Brassicaceae residues are also effective in weed management [79]. However, *Amaranthus retroflexus* (an opportunistic annual weed of many cropping systems) is reported to be one of the most challenging weed to control through ASD [19,80]. It appears that the weed control ability of ASD is due to phytotoxicity/phytotoxic volatiles generated by microbial activities.

Only six studies have been conducted to determine the effect of ASD on the crop yield. Korthals et al. [52] determined the effect of nine different treatments including ASD on crop yields of potato, carrot, and lily bulb and reported that ASD produced higher yields in all the crops compared to the untreated control. However, Di Gioia et al. [81] reported that ASD had no significant effect on tomato yield when composted poultry litter (22 Mg ha^−1^) and molasses (13.9 and 27.7 m^3^ ha^−1^) were used as the C sources. Nevertheless, plant nutrients such as potassium, calcium, magnesium, and iron accumulation had improved in ASD treated plants. Yield improvement might have resulted due to the combined effects of disease control, weed control, and improved soil nutrients. 

### 3.6. Mechanism of ASD 

**Figure 4:**
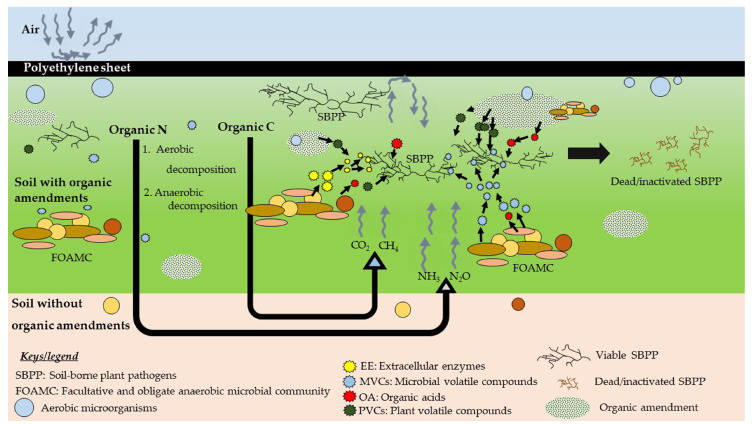
Proposed pathogen control mechanism(s) (simplified) during ASD. The decomposition of organic amendments is initiated by the activities of aerobic microorganisms (e.g., *Bacillus* spp.). Later, the growth of anaerobic bacteria (e.g., *Clostridium* spp.) is stimulated with the depletion of oxygen, and anaerobic decomposition of organic matter is initiated. This pathway is more complex and less energy demanding than that of the aerobic decomposition. Facultative and obligate anaerobic microbial communities (FOAMC) decompose the added organic C and produce several gases such as CO_2,_ CH_4,_ and volatile compounds. Decomposition of organic N leads to produce soil ammonium (NH_4_^+^) via mineralization. Finally, due to series of activities, CO_2,_ CH_4,_ N_2_O, and NH_3_ are released, and these gases have toxic effect on living matter. Combined effects of above released gases along with organic acids (OA), microbes released extracellular enzymes (EE), microbial volatile compounds (MVCs), and plant volatile/non-volatile compounds (PVCs) along with the change of soil physical properties may cause the inhibition of soil-borne phytopathogens (SBPP).

Only 34% of studies have reported the mechanism of ASD. Nevertheless, the exact mechanism of ASD is still not clear, and further studies are necessary. In ASD, the use of different carbon sources helps boosting soil microbial biomass and enzyme activities [54]. Covering with a plastic trap as well as the utilization of available oxygen by the aerobic microorganisms ultimately create an anaerobic soil condition. Figure 4 shows possible soil pathogen control mechanism(s) by ASD. 

Polyethylene sheets prevent further penetration of oxygen to the treatment creating a conducive environment for anaerobic microorganisms (e.g., Clostridial species). These anaerobic decomposers use C source to respire while releasing toxic anaerobic by-products such as CO_2_, NH_3_, H_2_S, CH_4_, and N_2_O [19]. However, these by-products are released to the atmosphere quickly, as soon as the tarp is removed or the holes are punched [79]. Researchers predicted that the limitation of oxygen along with the trapping of toxic compounds and lowered pH could control soil-borne phytopathogens [44]. Under the flooded conditions, microbes decompose liable C sources and release gases (or by-products) suppressing some of the phytopathogens [19]. ASD has shown significant changes in the whole soil microbial communities [46,65]. Mowlick et al. [82] reported the changes in microbial community structures (through clone library analysis) after ASD treatment. They observed ASD caused a reduction in diversity of bacterial communities of various phylogenetic groups and a domination of anaerobic clostridial class bacteria.

In ASD, accumulation of various volatile compounds with the potential to control phytopathogens greatly depends on the C source used [50,55,66,83,84]. In addition to pathogen control, plant growth promotion abilities of microbial volatile compounds (MVCs) have also been extensively reported [84,85,86]. These volatile compounds spread through soil by diffusion, and efficacy of volatile compounds is greater than non-volatile compounds [87,88,89]. Compared to the other MVCs such as enzymes, antibiotics, and toxins, microbial organic volatiles are typically small in size (up to 20 carbon atoms) with molecular mass ranging from 100 to 500 Daltons [85]. MVCs have a good diffusing ability under normal temperatures and pressures [90]. Volatile compounds produced by the bacteria are dominated by alkenes, alcohols, ketones, terpenes, benzenoids, pyrazines, acids, and esters, while fungal volatiles are dominated by alcohols, benzenoids, aldehydes, alkenes, acids, esters, and ketones [85]. Antifungal compounds such as dimethyl disulfide, dimethyl trisulfide, and acetoin are well reported [90]. Studies found that the fungal species such as *Aspergillus giganteus*, *Fusarium oxysporum*, *Penicillium viridicatum*, *Trichoderma viride*, and *Zygorhynchus vuilleminii* have abnormal morphologies in their conidiophores and hyphae when exposed to VOCs from bacteria and actinomycetes [91]. Rather than the production of volatile compounds [48], soil anaerobic bacterial communities could kill the phytopathogenic fungi through extracellular enzymes such as 1,3-glucanase and chitosanase, whereas obligate anaerobic *Clostridium beijerinckii* could suppress the spinach wilt fungi, *F. oxysporum* and *F. spinaciae* [61]. However, the prevailing groups of the microorganisms may be different based on the type of C source used and the treatment period of ASD, since some microorganisms are responsive to fluctuating redox potentials [45,92]. 

## 4. Challenges and Potentials of ASD as a Game Changer in the Tropics 

The world’s population is estimated to reach over nine billion by 2050, which is roughly 34% higher than it is today, although the carrying capacity is just seven billion [93]. It is also predicted that the rapid population growth in the tropics and in developing countries is mainly responsible for this increase, and agriculture should be revolutionized to meet the increasing food demand [93,94,95]. It has been projected that future food production cannot be predicted by the historical grain yield patterns, and relative rate of grain yield may decrease in the future. In other words, yield gain has plateaued over time [96]. Therefore, it is obvious that developing countries, especially those in the tropics, might be adversely affected [97,98]. On top of that, 20–40% of worldwide agricultural crop productivity has been affected by pathogen and pest attacks and weeds, causing a considerable economic loss [99]. For example, in India, annual crop loss could reach up to USD 19 billion [100]. In addition to crop loss, arable land degradation is also a global concern. With industrialization and exponential population growth in the tropical developing countries, reduction of arable lands is inevitable, and it is the biggest threat to agricultural productivity. In addition, small-scale agricultural systems, multiple cropping systems, year-round crop availability, high level of crop diversity well as pathogen diversity, poor use of technology, low agricultural literacy among farmers, and low mechanization are some of the common characteristics of tropical, specially developing world agriculture [101]. Therefore, agricultural productivity in developing countries located in the tropics seriously lags behind than that of the temperate countries [102]. Hence, not only modernizing the agriculture but also soil health should be taken into account. 

Interestingly, a majority of ASD studies have been carried out in temperate and sub-tropical regions. Of the studies reviewed, 63.3% were carried out in the USA. However, it has not been sufficiently applied or tested in the tropical regions, especially in the developing parts of the world where the agricultural system is completely different yet is the major income for a majority. Only one ASD study has been conducted each in Sri Lanka and in Nepal, and no records on ASD were found in other developing countries. The only ASD study conducted in Sri Lanka was published by our research group [55] and tested the ability of controlling soil-borne fungal pathogen, *Sclerotinia sclerotiorum,* using cabbage (*Brassica oleracea*) and leek (*Allium ampeloprasum*) cull piles, durian (*Durio zibethinus*) peels, and grass cuttings (*Axonopus compressus*) as C sources. During this pot assay, 60–100 mg g^−1^ of cabbage and leek cull piles were found to be effective in 100% mitigation of sclerotial germination. With the promising results of pot assay, field trials were conducted in Sri Lanka using leek, cabbage, and a mixture of leek and cabbage cull pieces at different rates as wet and dry applications. The highest mean sclerotial germination inhibition (96.66%) was associated with the application of wet leek cull pieces at the rate of 43.05 t ha^−1^ and dried cabbage cull pieces at the rate of 32.28 t ha^−1^ [103]. In a study conducted in Nepal, Bhandari et al. [104] reported the best control of clubroot disease of cauliflower caused by *Plasmodiophora brassicae* was achieved by the amendment of cheuri cake (*Diploknema butyracea*) as the C source, while molasses and rice bran treatments were ineffective. In addition, cheuri cake also increased the yield compared to the untreated control. Therefore, it seems that ASD with different C sources is a promising approach to achieve disease suppression and gain yield and improve soil condition in tropical soil and in developing countries as well. However, thorough studies are necessary targeting the tropical region since there is a severe information gap. One major limitation in ASD is the cost associated with the use of plastic/impermeable sheets, and it is estimated that ASD costs more than chemical fumigation [105,106]. In addition, labour cost and non-biodegradability of polyethylene are serious issues. However, when considering the long-term effects, it is a worthwhile investment, and polyethylene sheets can be reused/shared among farmers due to small scale agriculture. We found that, in traditional agricultural practices in Sri Lanka, farmers used to draw certain patterns on the ground near the fields to get vermivorous and insectivorous birds’ attention to the field so that pest attacks could be minimised (personal communication, traditional farmers in Dambulla and Polonnaruwa, Sri Lanka). With this information, we propose to use cover material with various patterns as another dimension to the ASD research. However, the higher cost of plastic and labour appear to be the major limitations in popularising ASD among low-income farmers in the tropics. Therefore, further research in search of biodegradable or durable and low-cost mulch is a must in order to popularise ASD in the tropics, especially in the developing world. However, application of ASD to the high-value crops such as strawberries and greenhouse grown tomatoes rather than the low-value crops such as spinach, banana, and eggplant may give a considerable income to the farmers. Finally, high quality research on ASD in the tropics should be extensively carried out. 

## Figures and Tables

**Table 1:** **Details of** successful ASD experiments conducted during the past few years.

C Source	Application Rate of C Sources (t ha^−1^)	Pathogens Suppressed	Mean Soil Temperature/Range (°C)	Treatment Period	Crop	Field/Greenhouse	Country	Reference
Fresh broccoli (*Brassica oleracea*)	34,38	*Fusarium oxysporum*, *Rhizoctonia solani, Verticillium dahliae*	25–32, 29–39	15 weeks	N/A	Field, plot	Netherlands	[44]
Perennial ryegrass (*Lolium perenne*)	40	*Fusarium oxysporum*, *Rhizoctonia solani, Verticillium dahliae*	25–32, 29–39	15 weeks	N/A	Field, plot	Netherlands	[44]
Grass or potato haulms	30	*Ralstonia solanacearum*	N/A	6 weeks	Potato	Laboratory, field	Netherlands	[46]
Wheat bran	2	*Meloidogyne incognita*	35.0	24 days	Tomato	Greenhouse, plot	Japan	[47]
Cereal rye (*Secale cereale*)	0.134	*Rhizoctonia solani*	20.8	4 weeks	Tomato, bell pepper	Field, plot	USA	[49]
Mustard (*Brassica juncea*) seed meal	4.9	*Rhizoctonia solani, Pythium ultimum, Fusarium oxysporum*	18–24	2 weeks	Apple	Growth chamber, pot	USA	[50]
Grass residues	40.0	*Rhizoctonia solani, Pythium ultimum*, *Fusarium oxysporum*	18–24	2 weeks	Apple	Growth chamber, pot	USA	[50]
Rice bran	20	*Verticillium dahliae*	21–23	4 weeks	Strawberries	Field	USA	[50]
Radish roots	100	*Fusarium oxysporum*	33.1	3 weeks	Spinach	Greenhouse, field	Japan	[48]
Mixture of fresh rye-grass species	50	*Verticillium dahliae, Pasteuria penetrans*	N/A	12 weeks	N/A	Field	Netherlands	[48]
Mustard (*Brassica juncea*)	50	*Fusarium oxysporum*	33.1	3 weeks	Spinach	Greenhouse, pots, field	Japan	[48]
Wheat bran	20	*Fusarium oxysporum*	33.1	3 weeks	Spinach	Green house, pots, field	Japan	[48]
Rice bran	4.4	*Rhizoctonia solani*, *Pratylenchus penetrans*	18–24	2 weeks	Apple	Growth chamber, pot	USA	[53]
Fresh orchard grass residues	20	*Rhizoctonia solani*, *Pratylenchus penetrans*	18–24	2 weeks	Apple	Growth chamber, pot	USA	[53]
Mustard (*Brassica juncea*) seed meal	4.4	*Rhizoctonia solani*, *Pratylenchus penetrans*	18–24	2 weeks	Apple	Growth chamber, pot	USA	[53]
Rice bran	20	*Phytophthora nicotianae*	15–35	4 weeks	Pepper	Field	Spain	[54]
Rapeseed cake	20	*Phytophthora nicotianae*	15–35	4 weeks	Pepper	Field, plot	Spain	[54]
Grape pomace	40	*Phytophthora nicotianae*	15–35	4 weeks	Pepper	Field, plot	Spain	[54]
Rice bran	20	*Fusarium oxysporum*	18–24	15 days	Strawberry	Growth chamber, pot	USA	[69]

N/A: not available or not reported.

## Data Availability

Data available on request.
